# Association of Intracranial Aneurysms with Meningiomas, Pituitary Adenomas, and Gliomas: Review of Possible Interrelationships

**DOI:** 10.1155/2013/383425

**Published:** 2013-09-15

**Authors:** Kevin Spitler, Doniel Drazin, George Hanna, Ashish Patel, Ray Chu

**Affiliations:** Department of Neurosurgery, Cedars-Sinai Medical Center, Los Angeles, CA 90048, USA

## Abstract

Asymptomatic intracranial abnormalities are increasingly becoming a focus of attention with the utilization of high-resolution imaging. The concurrence of tumor and aneurysm has been described, largely, by case reports and single-surgeon experiences. Recent papers have outlined the ethics of incidental findings and possible treatment algorithms. Incidental finding of an aneurysm occurs most commonly in patients with meningiomas, pituitary adenomas, and gliomas. Such an association may explain the mechanisms of aneurysm formation, growth, and rupture in acromegalic patients; however, insufficient data are available to link aneurysm with either glioma or meningioma.

## 1. Introduction

The radiographic characterization of a primary lesion may present a new clinical issue when a second asymptomatic intracranial lesion is identified. This issue is expected to become increasingly more frequent as the resolution of neuroimaging progresses [1, 2]. The ethics and clinical approach algorithm is currently under discussion [3]; however, the relationship between two concurrent intracranial lesions is not completely understood. The existence of a single lesion has been hypothesized to initiate additional lesions in the brain [4]. The occurrence of tumor together with aneurysm has been noted in case reports and series, but a comparison between tumor types and aneurysm location has been rarely studied.

Our focus is to document the concurrence of aneurysm and tumor across the literature and to describe the treatment outcomes, including observation. We hope to achieve a new understanding of the possible associations of meningiomas, pituitary adenomas, and gliomas with aneurysm formation, growth, and rupture. 

## 2. Methods

### 2.1. Literature Review

Articles were identified from a systematic literature review using PubMed and MEDLINE databases for the years 1950 through 2012 with the following search terms: “cerebral aneurysm,” “intracranial aneurysm,” “brain tumor,” “meningioma,” “glioma,” “glioblastoma multiforme,” “pituitary adenoma,” “ruptured,” “unruptured,” and/or “subarachnoid hemorrhage.” Additional publications were located and reviewed based on the references in the articles obtained from the database searches. Inclusion criteria required that appropriate articles be published as full-length articles in a peer-reviewed, English language journal. Studies were excluded if the co-occurrence of tumor and aneurysm could be separated. For example, aneurysms were excluded which appeared to develop within a postoperative area or that were labeled as pseudoaneurysms. If multiple published reports from a same study cohort were available or referenced within a larger review article, we included only the one with the most detailed information for both outcome and cooccurrence.

### 2.2. Statistical Analysis

All descriptive statistics were calculated using JMP 7.02 (SAS Institute). Averages for age, gender, type of tumor, rupture versus unruptured, location of aneurysm, and outcomes were calculated. 

## 3. Results 

Twenty-one articles were obtained from the search terms “intracranial aneurysm” and “brain tumor” on PubMed/MEDLINE databases. The review was expanded by exchanging the query term “brain tumor” for “meningioma” (199 articles), “glioma” (70 articles), and “pituitary adenoma” (33 articles). Two separate authors (D. Drazin and K. Spitler) reviewed the abstracts from the acquired articles, with 323 articles receiving detailed review.

 Of the 323 reviewed articles, 305 articles failed to meet the inclusion criteria and were excluded from the analysis. The subsequent analysis included the remaining 18 articles. The review focused on methodology, location and type of tumor and aneurysm, type of treatment, number of patients, mean patient age, and when available clinical outcomes data.

**Figure 1:**
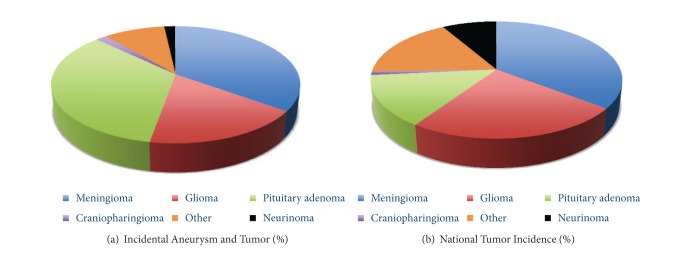
Concurrence of tumor and aneurysm by tumor type. (a) The pie chart illustrates the sum of reported concurrence of tumor aneurysm from the literature review. Pituitary tumor concurrent with aneurysm (34.2%) was more frequent in the review of the literature than the occurence of pituitary tumors alone in the national database (14.1%). From the greatest to the least, concurrent tumor and aneurysm identified were meningioma (34.9%), pituitary (34.2%), and glioma (17.8%) versus the incidence of tumor alone (b) which is estimated as meningioma (35.5%), glioma (23.9%), and pituitary (14.1%) (http://cbtrus.org/ and [5]).

**Figure 2:**
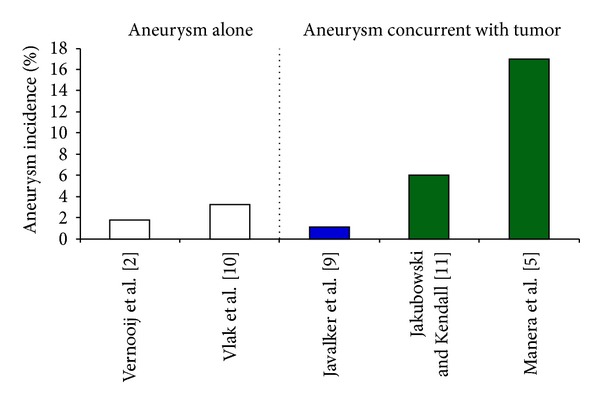
Incidence of asymptomatic aneurysm alone versus concurrence of asymptomatic aneurysm with symptomatic brain tumor. The percentage of subjects with aneurysm is plotted for references that provide total count statistics. The percentage of subjects with asymptomatic aneurysm alone (white bars) is similar to the percentage of subjects with asymptomatic aneurysm concurrent with brain tumor (meningioma, blue bar), with the exception of pituitary tumor with acromegaly (green bar [6]), which is approximately 4 times the upper limit of the former. References are provided in brackets.

Of the 18 studies identified that specifically included tumors and aneurysm, six were case reports, while the rest of the studies were retrospective case series. A total of 286 patients with a mean age of 49.9 years (13.6 SD) were reviewed. Descriptive statistics for the tumor and aneurysm co-occurrence were for meningioma (101), glioma (50), and pituitary adenoma (98) (Tables 1, 2, and 3, resp.). Additionally, 37 cases with concurrent aneurysm and tumor of other types were included (craniopharyngioma, primary CNS lymphoma, etc.) (Figure 1). Most of the aneurysms were unruptured (71.9% meningioma, 77% glioma, and 93.7% pituitary adenoma) and were located on the internal carotid artery (ICA). Additionally, to determine the prevalence of intracranial tumors, we examined several large case series of incidentally discovered asymptomatic intracranial tumors. With the exception of acromegalic patients [6], as discussed in the following, the coexistence of aneurysm and tumor in our review of the literature appears to be consistent with coincidence (Figure 2). 

## 4. Discussion

With the increasing usage of sophisticated neuroimaging techniques such as MRI and CT angiographies, the discovery of either incidental intracranial neoplasms or aneurysms has increased. The impact of these incidental lesions on a patient's health has varied in significance and has yet to be elucidated. 

### 4.1. Incidental Finding of Meningioma and Aneurysm

Meningioma and aneurysm were the most common combination in the series of Pia et al. [4] (29%) and in our review as well. Several of Pia's hypotheses [4] for a causal relationship between tumor and aneurysm continue to be evoked, especially dysgenetic and hormonal etiologies. Additionally, these tumors are often described as vascular with the suggested casual mechanisms of proangiogenic factors and/or increase in regional blood flow; yet, ipsilateral tumor and aneurysm were found in only 76% of cases [7]. Despite these interesting features, several arguments support the coincidental nature of the concurrence. The female predominance was 78% in our review of the literature, similar to the 8 : 1 ratio of women to men generally reported for meningioma alone [8]. The low frequency of the concurrence of meningioma and aneurysm in multiple series over the last 60 years continues to support a coincidental mechanism; thus, the contribution of estrogen and dysgenetic factors appears to be minor when an additive relationship may be expected. Despite several publications on the concurrence [9], few give a total number of cases of meningiomas without aneurysm to provide an understanding of the true incidence; one exception is Javalkar's group [10], who recently described 5 cases in their series of 426 meningiomas (1.1%). An estimate of 1% of tumors with associated aneurysm is less than the predicted prevalence of unruptured aneurysm rate in patients without comorbidities, which in a meta-analysis was 3.2% [11] and on neuroimaging of asymptomatic individuals was 1.8% [2]; thus, the coexistence of aneurysm and meningioma in our review of the literature appears to be consistent with coincidence. 

### 4.2. Incidental Finding of Pituitary Adenoma and Aneurysm

The series of Jakubowski and Kendall [12] found high incidence (6%) of aneurysm on review of pituitary adenomas and craniopharyngiomas. Notably, 4 of their 11 cases had acromegaly and after their removal, the percentage was recalculated at 3.9%. In 10 of 11 cases, the aneurysm was on vessels adjacent to the adenoma. This study reported exclusive anterior circulation aneurysms; however, four-vessel angiography was not always performed, and thus, this increased prevalence of concurrent aneurysm and tumor may be even greater. In contrast, the series by Pant et al. [13] found 40% of aneurysms to be distant from pituitary adenoma. They dismissed the acquisition of four-vessel cerebral angiography as beneficial for transsphenoidal surgery and suggested noninvasive structural MRI and MRA imaging for presurgical planning.

The limitation of selective angiography may be appreciated in light of Manara's study [6] of acromegalic patients. Seventeen percent of their series had at least one incidental aneurysm diagnosed on MRA. Their study provided support for a causal relationship by a positive correlation of aneurysm and growth hormone measurement in acromegaly.

### 4.3. Incidental Finding of Glioma and Aneurysm

Pia et al. [4], Licata et al. [14], and Taylor [15] have the largest case series of glioma and aneurysm concurrence. Pia's series [4] reported a 73% mortality in the glioma group, which is greater than the 40% overall mortality in their series for tumor and aneurysm coexistence in general. Likewise, in the 2 cases of concurrent glioma and aneurysm in Taylor's series [15], the patients died at 2 and 28 days after surgery. Another case described treatment of the tumor alone with subsequent SAH, but with good outcome [16]. 

With more recent techniques, Licata et al. [14] approached 8 gliomas with an aneurysm with no change in the prognosis over gliomas treated alone. Of their 8 cases, there was 1 case of perioperative death secondary to pulmonary embolism. Otherwise, the cases improved or remained stable, and patient survival ranged from 7 months to 17 years (median 1 year). Aneurysms were clipped in the 2 cases that presented with SAH. The remaining aneurysms were treated conservatively without subsequent rupture in the setting of either conservative or partial removal of glioma. Overall, their treatment algorithm proposed a combined operation if feasible, as permitted by the logistics of the tumor alone. 

In cases where the tumor is distant from the aneurysm, clipping or endovascular treatment can be attempted prior to tumor resection to avoid potential destabilization of the aneurysm [17]. Successful combination surgery for ruptured aneurysm and tumor resection has been documented with patient followup for 22 months [18, 19]. An additional case described the surgical treatment of aneurysms and grade II astrocytoma in a single operation with good outcome and 6 month followup [20]. 

In regards to a causal relationship, Pia et al. [4] found the aneurysm to be ipsilateral to tumor location in all cases in which the data were reported. Likewise, Licata et al. [14] reported a contralateral aneurysm in only 1 of 8 cases. The caveat in these two series is the use of ipsilateral angiography only in the diagnostic workup. A systematic approach in a large case series is required to address the possibility of a causal angiogenic relationship between glioma and aneurysm. Additionally, although the total number of brain tumors is reported by both Licata et al. [14] and Pia et al. [4], the percentage of gliomas concurrent with aneurysm versus solitary gliomas is not given by either author, preventing further evaluation of a causal relationship between glioma and aneurysm. Until such data becomes available, the relationship of glioma and aneurysm cannot be considered more than an incidental finding. 

Patients who received early resection of their low grade glioma had a survival advantage compared with conservative management, in recent cohort studies [21, 22]. As patients may become increasingly motivated to choose surgery based on these data, the question of screening for aneurysms arises. The long-term outcome of the patient may improve if asymptomatic lesions are addressed at the time of initial surgical planning; however, as described above, it is unclear at this time the prevalence of concurrent glioma and aneurysm. A recent article analyzing the rationale of screening for intracranial abnormalities as a means of preventive neurosurgery concluded that there was justification for screening for aneurysms whose endovascular interventions had low treatment-associated morbidities, while there was no justification for screening for asymptomatic tumors [23]. There is no universal method of addressing incidental intracranial findings on brain MRI; however, as knowledge of the interaction between tumor and aneurysm increases, it is expected that evidence-based algorithms can be generated. In the interim, protocols have been discussed regarding therapy for asymptomatic incidental findings [3, 24–26].

## 5. Outcomes

Better outcomes in recent reports of treatment of aneurysm and tumor are attributed to preoperative endovascular coiling, combined aneurysm clipping and tumor resection in one sitting, and microsurgical techniques [17–19]. The use of MRA may be justified in the workup of patients with acromegaly due to the increased concurrence of aneurysm [6]. Other tumors have coexisting aneurysms as incidental findings and four-vessel angiography may be pursued to characterize the location and number of aneurysms [20, 27, 28]. In patients who did not consent to aneurysm clipping or coiling, tumor resection has been performed alone with good results [7, 10].

A portion of small aneurysms progresses to subarachnoid hemorrhage, and asymptomatic aneurysms need to be evaluated considering patient risk factors (age, history of hypertension, and location) independently of tumor, during tumor surveillance [29]. The reports reviewed are biased toward the patient when either tumor or aneurysm was symptomatic at presentation (and then the other was discovered to be coexisting as an incidental finding) with surgery then performed to relieve patient symptoms without tracking the growth of the asymptomatic lesion over 6-months.

Aneurysm rupture is less commonly the presenting symptom (22–45% [4, 14]) with incidental tumor discovery; therefore, the clinical team will more frequently need to discuss the risks and benefits of aneurysm interventions. Ideally, incidental aneurysms should be treated prior to surgical intervention for tumor when the aneurysm is large, has characteristics worrisome for rupture and is amenable to endovascular or microsurgical treatment. In recent case reports, surgical treatment of tumor and aneurysm accomplished in a single operation had good outcomes [14, 18–20]. Taylor's report of SAH complications when tumor resection is performed without respect to aneurysm suggested that tumor removal may destabilize incidental aneurysms adjacent to the tumor bed [15]. Taylor [15] describes 2 cases that presented with signs of increased ICP, with preoperative angiograms negative for aneurysm. These patients were taken to surgery for tumor removal, and although no aneurysms were noted, there was difficulty with hemorrhage during tumor resection. Both patients improved postoperatively until signs of subarachnoid hemorrhage occurred on days 12 and 28, for cases 1 and 2, respectively. Postoperative angiograms were reported to be positive for saccular aneurysms that are likely now better categorized as traumatic pseudoaneurysms. Recent reports have not found this to be a frequent occurrence, which may be attributed to improved imaging modalities obtained preoperatively. Thus, in situations where the aneurysm is distant to the tumor, discussion with the patient of the risks and benefits based on their general risk factors for aneurysm rupture may be had without additional calculation for coexistence of intracranial tumor [17]. 

## 6. Conclusions

In general, the concurrence of intracranial tumor with aneurysm is similar to the observed prevalence of unruptured aneurysm in subjects without comorbidities [27]. When intracranial tumors were examined by type, pituitary tumors associated with acromegaly may have an increased coexistence of aneurysm and tumor, especially in the anterior circulation aneurysms [6]. In the acromegalic patient, angiography is expected to be relevant to surgical planning. Limitations of the available data are due to the infrequency of studies that performed four-vessel angiography as well as the small number of studies that reported the total number of the tumor type identified during the study period at the institution. Without these data, it is possible that the concurrence of intracranial tumor and aneurysm is underreported. The review of the literature does not promote routine investigation for aneurysm when an intracranial tumor is identified in the clinical setting; however, the review does identify opportunities to expand knowledge of intracranial abnormalities as the limitations described above can be addressed with institution brain tumor databases and noninvasive four-vessel angiography.

## Figures and Tables

**Table 1:** Demographic and clinical characteristics of patients with aneurysm and meningioma.

Characteristics	Number	(%)
Total number of patients	101	
Patient age in years mean (SD)	51.2 (14.4)	
Female	59	
Initial presentation with sympathology due to tumor	56/78	72
Aneurysm location		
ICA	30	36
MCA	4	5
ACOMM	17	20
ACA	7	8
Basilar	2	2
PCOMM	5	6
Multiple	19	23
Not reported	17	
Ipsilateral aneurysm	63/86	73
Unruptured aneurysm	71	71
Outcome		
Good recovery	49	85
Died	9	15
Not reported	43	

**Table 2:** Demographic and clinical characteristics of patients with aneurysm and glioma.

Characteristics	Number	(%)
Total number of patients	50	
Patient age in years mean (SD)	47 (12)	
Female	15	
Initial presentation with sympathology due to tumor	23/30	76
Aneurysm location		
ICA	15	34
MCA	12	27
ACOMM	7	16
ACA	6	14
Basilar	2	5
PCOMM	1	2
Multiple	1	2
Not reported	6	
Ipsilateral aneurysm	21/23	93
Unruptured aneurysm	38	77
Outcome		
Good recovery	12	35
Died	22	65
Not reported	16	

**Table 3:** Demographic and clinical characteristics of patients with aneurysm and pituitary adenoma.

Characteristics	Number	(%)
Total number of patients	98	
Patient age in years mean (SD)	50 (13)	
Female	42	
Initial presentation with sympathology due to tumor	90	92
Aneurysm location		
ICA	63	64
MCA	11	11
ACOMM	6	6
ACA	11	11
PCOMM	1	1
Multiple	6	6
Not reported	91	93
Unruptured aneurysm	91	93
Outcome		
Good recovery	84	84
Died	14	14
